# Characterizing Websites That Provide Information About Complementary and Integrative Health: Systematic Search and Evaluation of Five Domains

**DOI:** 10.2196/ijmr.9803

**Published:** 2018-10-10

**Authors:** Annie T Chen, Lisa Taylor-Swanson, Ronald W Buie, Albert Park, Mike Conway

**Affiliations:** 1 Department of Biomedical Informatics and Medical Education University of Washington School of Medicine Seattle, WA United States; 2 College of Nursing University of Utah Salt Lake City, UT United States; 3 Department of Software and Information Systems College of Computing and Informatics University of North Carolina at Charlotte Charlotte, NC United States; 4 Department of Biomedical Informatics University of Utah School of Medicine Salt Lake City, UT United States

**Keywords:** complementary and integrative health, online information quality, website quality, website content, acupuncture, massage, homeopathy, reiki, yoga

## Abstract

**Background:**

In recent years, there has been an increase in the utilization of complementary and integrative health (CIH) care, and an increase in information-seeking behavior focused on CIH. Thus, understanding the quality of CIH information that is available on the internet is imperative. Although there have been a limited number of studies evaluating the quality of websites providing information about specific CIH-related topics, a broad evaluation of CIH websites has not been conducted.

**Objective:**

This study was designed to fill that gap. We set out to assess website quality in 5 CIH domains: (1) acupuncture, (2) homeopathy, (3) massage, (4) reiki, and (5) yoga. This study aimed to 1) characterize the websites by type and quality; 2) evaluate website characteristics which may affect readers’ perceptions, specifically message content, structural features, and presentation style, and 3) investigate the extent to which harms, benefits and purposes of use are stated on websites.

**Methods:**

This study employed a systematic search strategy to identify websites in each of the target domains to be evaluated. The websites were then classified by type, and a set of checklists focusing on quality, message content, structural features, and presentation style was used to evaluate the websites. Lastly, we performed content analysis to identify harms, benefits, and perceived purposes of use.

**Results:**

There were similarities across domains regarding their overall quality and their message content. Across all domains, a high proportion of websites received strong scores in terms of ownership, currency, interactivity and navigability. Scores were more variable concerning authorship, balanced presentation of information and the use of sources of information. However, there were differences regarding their structural features and presentation style. Acupuncture and reiki sites tended to include more external links, and yoga, fewer. There was variation across domains in the extent to which the websites contained domain-specific terminology. Websites tended to provide an extensive list of potential benefits, while reporting of harms was scarce.

**Conclusions:**

This is the first study to perform a multidimensional assessment of websites in multiple CIH domains. This review showed that while there are similarities among websites of different CIH domains, there are also differences. The diverse distribution of website types suggests that, regardless of CIH domain, the public encounters information through many different types of media, and it would be useful to consider how the presentation of this content may differ depending on the medium. The characteristics for which variability exist are areas that warrant greater attention from researchers, policy makers, clinicians and patients. There is also a need to better understand how individuals may interact with CIH websites, and to develop tools to assist people to interpret the CIH-related information that they encounter.

## Introduction

Increasing numbers of people have sought CIH care in the US [1] and worldwide [2]. Complementary and integrative health (CIH) and complementary and alternative medicine (CAM) are often used to refer to nonmainstream health care practices. The term CIH is now being increasingly employed to emphasize that these complementary modalities are being used alongside, rather than in place of, conventional medicine [3]. In this article, we refer to CAM whenever the term was used in a cited information source; otherwise, we employ the term CIH.

The Centers for Disease Control reported that 38% of adults in the US used one or more CAM practices in a 12-month period [4]. CAM use has been reported to be as high as 87% among cancer patients [5]. Previous research has also reported that there is substantial use of CAM for particular conditions including lower back pain [6], gastrointestinal diseases [7], and cardiovascular disease [8]. Alongside this, an increasing number of people seek web content to inform decision-making. In 2009, 35% of internet users looked online for information about alternative treatments, up from 28% in 2002 [9]. People also access websites, forums, blogs, online communities, and social networks to discuss CIH [10].

Various factors contribute to patients’ use of CIH. First, patients seek health care that is more in line with their values, beliefs, and orientations. This may include a valuation of more holistic approaches to health, an orientation towards wellness, and a desire to participate in treatment decisions [11-17]. Relying on personal experiences [16,18-20], use of social networks for information and decision making [17,19-22], and social relationships may also influence individuals’ decisions to seek CIH care [23-26]. Lastly, dissatisfaction with conventional practitioners and the lack of effectiveness of conventional treatments may also influence this shift towards CIH [14,20,27-29,17].

Given the increasing utilization of CIH modalities and increase in CIH-related information seeking, understanding the quality of CIH information that is available on the internet is imperative. In recent years, there have been concerns about the quality of CIH-related information on the internet, including attention to and concern about CIH-related news coverage [30-32]. Additionally, a review of randomized controlled trials for CIH has reported that the quality of safety reporting needs improvement [33].

However, to date, there have been few studies of the quality of CIH online health information. Those that have been conducted focus on the evaluation of websites presenting CIH information relating to specific topics, such as the utilization of CIH for cancer [34,35], and ginseng, ginkgo biloba, and St John’s wort[36]. For example, one study of 150 websites containing information on ginseng, ginkgo biloba, and St John’s wort reported that most CIH websites had poor technical criteria compliance (authorship, references, editorial process) [36]. Additionally, websites often contained information that could result in harm if acted upon and omitted vital information. In another study of websites selling St John’s wort, websites with references to information sources were more likely to give correct indications and to list drug interactions [37]. A study of websites of National Cancer Institute-designated comprehensive cancer care centers reported that, while more sites are providing information about CIH modalities, the quality and ease of navigability of these sites was variable [34]. Existing research has also examined the portrayal of CIH information in other media including Wikipedia and news media. It has been concluded that 56% of the reviewed articles in Wikipedia needed substantial improvements in content [38]. Also, there is considerable variability in news reporting practices about CIH, and much of the information that the public receives is inaccurate and incomplete [30].

When we consider the increased public interest and consumption of CIH against this backdrop of concern regarding online CIH information, a clear need emerges to better understand the characteristics of CIH-related websites. This project was designed to fill that gap. To do so, we aimed to characterize websites based on multiple dimensions. First, we categorized websites by type and evaluated them regarding quality using a checklist based on extant literature [39,40]. Though website quality may influence users’ perceptions and inclination to use CIH, users may also be influenced by their perceptions of the credibility of these websites. Previous studies have identified various factors that can influence perception of website credibility, including the site type [41], design look [42], information structure [42], information focus [42], message content [43], and structural features such as the inclusion of a navigation menu, privacy policy, and links to external sites [43]. Thus, our evaluation of websites also considered characteristics that have been associated with perceived website credibility. Lastly, given the concern regarding safety and the potential for harm, we investigated the extent to which benefits, harms, and the purposes of websites were stated, to contextualize the information that was provided. This study had the following aims:

- Characterize the websites by their type and evaluate overall quality
- Assess the message content, structural features, and presentation style of websites that may influence the viewers’ perceptions apart from quality
- Examine the extent to which harms, benefits, and website purposes are stated

Given the great diversity of CIH modalities, we reviewed several domains to provide a broader characterization of the quality of online CIH information. Previous studies of CIH have employed a classification system with 5 types [5,30,34]. We selected 1 modality in each of the 5: acupuncture (whole systems), homeopathy (biologic), massage therapy (manipulative), reiki (energy medicine), and yoga (mind-body).

## Methods

### Overview

This study employed a systematic search strategy to identify websites in each of the target domains to be evaluated. The websites were then classified by type, and a quality checklist and other checklists of factors that may influence credibility were used to evaluate the websites. Lastly, the websites were coded to identify harms, benefits, and perceived purposes of use.

### Sample

#### Search Strategy

**Figure 1:**
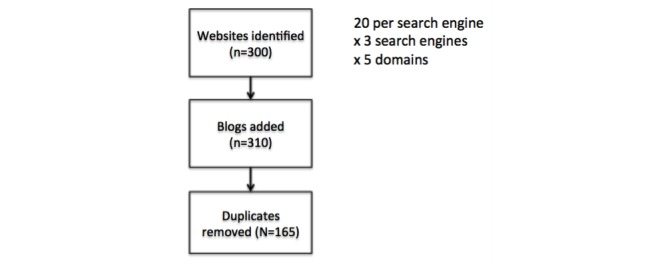
Search strategy.

A systematic search strategy was developed to identify websites with relevant content for each of the 5 domains. Three popular search engines [9] including Google, Yahoo, and Bing were used to search for the following terms “reiki,” “homeopathy,” “massage,” “acupuncture,” and “yoga.” People tend to look at the first page of the search engine results and occasionally go to the second search engine results page (SERP) [44], and SERPs yield an average of 8-10 number of sites [45]. Thus, the first 20 results, excluding ads, from each search engine were viewed and saved. This yielded 300 results (ie, the product of 5 domains searched, 3 search engines, and 20 results). Two blogs were added to the search results for each domain to ensure that they were represented in the websites that we examined. To do so, the search for each domain was rerun, adding the search term “blog”, and the first two appropriate entries added. Duplicates were omitted, yielding a total of 165 sites (Figure 1). Browser and cookie information was erased before conducting the searches, and sponsored links and advertisements were excluded. Websites that aggregate sources of information rather than provide information itself were excluded, including thumbtack.com and yelp.com.

#### Quantitative Content Analysis

Each of the research aims was based on quantitative content analysis, defined as a research method in which content is assigned to categories according to rules, and the analysis of relationships involving these categories is conducted using statistical methods [46]. Each website was viewed by at least one trained coder and evaluated for type, quality of online information, message content, structural features, and presentation style, per the criteria to be discussed in subsequent subsections. Thirty-five of the 165 websites (21.2%) were double-coded, and interrater reliability was evaluated by calculating a Cohen kappa statistic [47] within each domain and across domains. The Cohen kappa across all 5 domains was .82, indicating a high level of interrater reliability. The agreement for each of the individual domains is reported in Multimedia Appendix 1. After interrater reliability was calculated, the 2 coders discussed and resolved disagreements on the websites that were double-coded.

Each coder viewed the number of pages of a website necessary to evaluate it based on each of the evaluation criteria. Some websites may have required the coder to view every single page. For others, particularly extensive ones, it might not have been necessary for the coder to view every single page to evaluate the website for all criteria. However, to ensure consistency, both coders reviewed at least three pages for each website: “about,” “home,” and “contact.” In the case of blogs, coders answered the scale items based on the most recent blog entries (minimum of 10).

Due to the size of some of the larger websites, it was necessary to limit the amount of time that coders spent on reviewing them. Fifteen minutes was selected in order to standardize the procedure that coders followed. Coders noted if more than 15 minutes was necessary to evaluate and code a site correctly. In these cases, a code of “not present” does not necessarily mean the topic was not present, but rather, that it was not located within 15 minutes of reading and that it was hard to find. Twenty-four (14.5%) of the websites took longer than 15 minutes for a coder to review. Our reviews of media sites included not only the particular article that came up on our search but also other articles that were topically related and were available on the same website. Reading multiple articles ensured that our coding would reflect the overall media website content for the domain topic.

### Aim 1: Website Type and Quality

#### Website Type

We categorized websites according to a typology that has been presented by Sillence and colleagues [39]. This typology was comprised of 10 different types of health advice websites: (1) web providers and portal sites, (2) support groups, (3) charity sites, (4) government websites, (5) companies or clinicians promoting the sale of CIH services, (6) sales sites specific to CIH products, (7) personal sites such as blogs, (8) information services, (9) media sites, and (10) clinician sites. This schema was revised to make it more suitable for CIH-related subject matter, and to increase the inclusivity and clarity of the terminology used. We present the schema, along with sample websites classified into each category in the “Results” section.

#### Quality Assessment

To perform a general assessment of quality, we employed the Sandvik scale, a well-established quality scale originally developed for the analysis of urinary incontinence websites [42]. Since its development in the late 1990s, the scale has been applied to multiple domains including CIH [35,40,48-50]. It is comprised of 7 items (see Table 1), each rated as 0 (absent), 1 (partially present), or 2 (present).

In addition to the Sandvik instrument, we also considered many other instruments for assessing website quality including DISCERN, Journal of the American Medical Association, and Health on the Net certification. We ultimately decided that the Sandvik instrument, along with the other checklists that we describe in aim 2, were most appropriate to perform a well-rounded assessment of different factors that might influence a person’s interaction with a CIH website. In particular, we decided not to employ the widely used DISCERN instrument because many of the questions from DISCERN relate to communication of different treatment options for a given condition, and CIH-related websites are not necessarily disease-specific. Many CIH modalities focus on wellness and preventive health care practices, rather than treatment for a particular condition.

### Aim 2: Message Content, Structural Features, and Presentation Style

Aside from the characteristics that were examined in the Sandvik scale, other aspects of quality can also be related to users’ credibility perceptions and potentially, their subsequent intent to act upon the information presented by websites. In this aim, we characterize websites further based on 3 categories: (1) message content (inclusion of statistics, testimonials, and quotes), (2) structural features, such as the inclusion of a navigation menu, privacy policy, and links to external sites, and (3) presentation style (Table 2).

**Table 1:** Sandvik scale items and rating criteria.

Quality	Rating criterion
Ownership	2 (name and type of owner clearly stated on the “contact us,” “about” or similar page) 1 (other indications of ownership are present but hard to find, requiring several clicks through to various pages and not found on the contact/about pages) 0 (no indication of ownership)
Authorship	2 (author’s name and qualification clearly stated on the “contact us,” “about” or similar page) 1 (other indications of authorship are present but hard to find, requiring several clicks through to various pages and not found on the contact/about page) 0 (no indication of authorship)
Source	2 (references given to scientific literature) 1 (indications of source to nonscientific sources) 0 (no indication of source)
Currency	2 (date of publication or update clearly stated on all pages) 1 (indications of currency are not found on every page) 0 (no indication of currency, including timestamps based on standard templates for the entire website)
Interactivity	2 (clear invitation to comment or ask questions by an email address or link to a form) 1 (any other email address on the site) 0 (no possibility for interactivity)
Navigability	2 (information easily found by following links from the home page) 1 (information found only with difficulty by following links, search engine provided if information widely scattered on site) 0 (information scattered around, no search engine)
Balance	2 (offers balanced information) 1 (biased in favor of own products or services) 0 (only promoting own products or services)

**Table 2:** Message content, structural features, and presentation style definitions.

Website characteristic			Definition
**Message content**			
	Statistics	Pages with numeric information	
	Testimonials	Pages with stories, narratives or accounts	
	Quotations	Pages with a quote regarding the health topic/modality appearing in the main text of the website	
**Structural features**			
	Navigation menu	Pages with a menu or list that serve as a directory	
	Privacy policy	Pages with a link to the website’s privacy policy	
	Links to external sites	Pages that include a link to external websites	
	Physical address	Pages with contact information (address or phone number)	
	Third party	Pages with an accreditation or third-party endorsement (Health on the Net code or otherwise)	
**Presentation style**			
	Prominence	Is CIH^a^ information prominently displayed?	
	Visual appeal	Colors, images, and animation are pleasing and professional looking (high-quality website)	
	Advertisements	Absence of external advertisements for special offers and commercial sales	
	Terminology	Use of CIH language and terms specific to that domain (eg, “chi” or “Qi” for acupuncture, “like cures like” for homeopathy, “friction” for massage therapy, “Ki” for reiki, and “prana” for yoga)	

^a^CIH: complementary and integrative health.

The scales for message content and structural features were based on those used in a study investigating the effect of website characteristics on perceptions of website credibility [43]. The scale for presentation style was based on items that were included in a study of the perceptions of CIH information conveyed by websites of medical institutions [51]. These 3 scales were revised to prevent duplication of items among the instruments, to minimize the need for redundant coding. The items in each of these scales were assigned 1 of 2 codes: 1 (present), and 0 (absent).

#### Examining Associations Between Domain and Website Attributes

We employed inferential methods for contingency tables to examine associations between specific domains, and the scores that websites were assigned for quality characteristics, message content, structural features, or presentation styles. We conducted chi-square tests of independence to examine whether there was association between domain and the presence of external links, and domain and the use of specialized terminology. In the case of domain versus source attribution, due to the presence of expected frequencies of less than 5, we employed Fisher’s exact test [52]. A chi-square test of independence or Fisher’s exact test on its own merely shows that there is an association between 2 nominal variables; it does not show which cells are contributing to the lack of fit of the model [53,54]. To further examine the nature of the dependence, we calculated standardized Pearson residuals. A standardized Pearson residual that exceeds 2 in absolute value indicates a lack of fit of the model in a given cell [53,54]. Lastly, we followed up with pairwise chi-square comparisons using the approach recommended in Benjamini and Hochberg to control for the false discovery rate [55], and to further contextualize differences between the domains.

### Aim 3: Harms, Benefits, and Website Purposes

In previous CIH-related research, potential harms and benefits have often been a source of concern. For example, it has been reported that media coverage of CAM is inconsistent [30], and studies have argued for the need for increased awareness of harms and benefits [56]. Thus, we also investigated the extent to which harms and benefits were reported in our sample. We also noted the purposes of these websites to provide additional context regarding the nature of websites in each domain.

We reviewed literature on the 5 domains for harms and benefits. We started our coding scheme based on this literature and then added relevant codes from the process of coding the websites. Two coders independently coded the same 2 websites from each domain for harms, benefits, and purposes. They then came together to discuss and agree upon a coding scheme (Table 3). The remaining websites were then divided between the 2 coders, and each coder proceeded to code the rest of the websites that they were assigned. The code “misinformation” was applied to situations in which parts of the website text argued that misinformation caused by the modality had the potential to cause harm. For example, in one case, a yogi argued that Westerners should not be doing traditional yoga due to cultural differences in normal resting postures, such as sitting in chairs or on the ground, and that the expectation that yoga should be the same resulted in higher levels of injury among his advanced students. The websites were not mutually exclusive, meaning, that a website could indicate more than one harm, benefit, or purpose.

**Table 3:** Definitions of harms, benefits, and purposes.

Content dimension		Definition
**Harms**		
	Contamination	Person could be harmed by contaminated supplements
	Misinformation	Misinformation about or caused by the modality that has the potential to cause harm
	Harmful if improper	Harm could occur if the patient saw an untrained provider, or if a supplement was inappropriately tried without seeing a clinician
	Syncope	Mention of syncope
	Bleeding	Mention of bleeding
	Bruising	Mention of bruising
	Other harm	Other harm not noted in this schema
	No harm	No harm was mentioned
**Benefits**		
	Psychological	Any psychological symptoms (ie, anxiety, depression)
	Pain relief	The site discusses how a modality decreases pain or provides pain relief
	Circulatory	The site discusses how a modality improves circulation
	Neurologic	Any neurologic disease symptom (multiple sclerosis, dementia, neuralgia, paresthesia)
	Gynecologic	Any gynecologic disease or symptoms (eg, dysmenorrhea, menopausal transition symptoms, hot flashes, premenstrual syndrome)
	Digestive	Any digestive disease or symptom (eg, irritable bowel syndrome, Crohn’s disease, diarrhea, constipation)
	Endocrine	Any endocrine disease or symptom (eg, diabetes mellitus, Cushing's syndrome)
	Cardiovascular	Any cardiovascular disease or symptom (eg, congestive heart failure, palpitations, tachycardia)
	Posture	Any mention of benefits for posture
	Multiple benefits	More than one benefit was mentioned or the website stated that there were multiple benefits
	No benefit	No benefits were mentioned or the website stated that there were no benefits
	Other benefit	Other benefit mentioned that does not fit the categories listed
**Website purpose**		
	Information	The site provides information for the public about a modality
	Training	The site provides training to clinicians and teachers of a modality
	Sales	The site offers sales of services or products
	History	The site provides historical context regarding the modality
	Quality	The site provides evidence or information regarding the quality of a modality, or how to locate a quality provider, supplement or class
	Professionalization	The site provides information regarding the professional status of a modality or aims to corroborate the professional nature of the modality
	Promote research	The site promotes research on a modality
	Present learned consensus	The site presents consensus of clinicians or teachers of a modality
	Other motivations	Any other motivation not listed

## Results

### Sample

Based on the procedure described in the “Methods” section, 165 websites across the 5 CIH domains of acupuncture, homeopathy, massage, reiki, and yoga were selected for this review. The domains, their size, and example sites, are presented in Table 4. As can be seen, this review included websites that were specifically focused on a domain, as well as websites with a more general focus that include content relating to CIH domains.

### Aim 1: Website Type and Quality

#### Website Type

We classified websites by type, such that websites could be considered as having more than one type (Table 5). For example, a website could simultaneously be coded as a clinician's website, a blog, and promoting the sales of products and services. Almost half of the websites reviewed were classified into more than one type (74/165, 44.8%), and approximately one-fifth (34/165, 20.6%), into two or more types.

**Figure 2:**
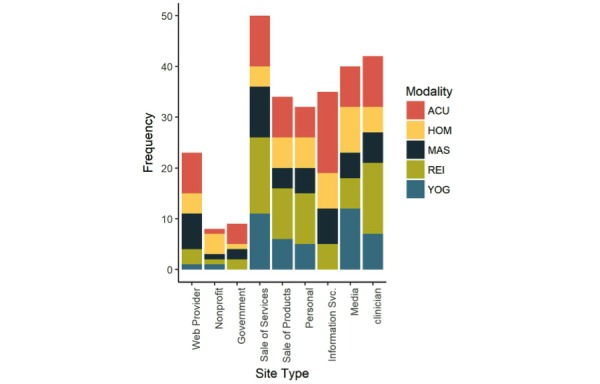
Modalities by site type. ACU: acupuncture, HOM: homeopathy, MAS: massage, REI: reiki, YOG: yoga.

With respect to website categories across the 5 domains, there were no support sites and few sites that were classified as government (9/165, 5.4%) and nonprofit organizations (8/165, 4.8%) as shown in Table 6 and Figure 2. The most common government source of information was the National Center for Complementary and Integrative Health (NCCIH). Overall, sites promoting the sale of services (50/165, 30.3%) were the most common. High frequencies of clinician sites (42/165, 25.4%), media (40/165, 24.2%), information services (35/165, 21.2%), and sales of products (34/165, 20.6%) were also observed.

**Table 4:** Number of websites by domain, along with examples (N=165).

Domain	Example	Sites, n (%)
Acupuncture	American Academy of Medical Acupuncture	37 (22.4)
Homeopathy	Homeopathic Educational Services	33 (20.0)
Massage	“Massage Therapy Styles and Health Benefits” article on WebMD	31 (18.8)
Reiki	“What is Reiki” article on US News and World Report website	37 (22.4)
Yoga	“Yoga” page on Wikipedia	27 (16.4)

**Table 5:** Definitions and examples of website types.

Website type	Definition	Examples^a^
Web providers and portal sites	Information and advice supplied by web provider rather than a physical organization Portals act as catalogs of information providing a gateway to many other sites providing information and advice	Medical News Today Acupuncture.com: Gateway to Chinese Medicine, Health and Wellness
Support groups	Often run by individuals or on behalf of support groups May be local, national or global in scale Often contains forums where consumers can read comments and contribute to discussions	None
Nonprofit organizations	An organization that is aimed at providing a service that is free and not for profit	National Center for Homeopathy Society of Homeopaths
Government websites	Provide patient information in the form of news, features and fact sheets	National Center for Complementary and Integrative Health Medline Plus
Companies or clinicians promoting sales of CIH^b^ services	A corporate, institutional, or clinician website that promotes the sale of its own services	University of Maryland Medical Center Simply Hot Yoga Wellness Center
Sales sites that include the sale of CIH products	Sales sites promote and sell drugs, medical devices or health plans often in addition to providing information	mindbodygreen 1-800 Homeopathy
Personal sites (blogs)	Contains personal experiences of illnesses and health issues	Quackwatch Homeopathic Medicine Blog
Information services	Websites that provide articles on health and illness issues	Science-Based Medicine WebMD
Media sites	Extensions of print or television media sites that provide the latest news and commentary on health features	Vanity Fair Reader’s Digest Best Health
Clinician sites	Information on specific health issues or specialist clinics run by medical professionals	To protect the privacy of the practitioners, examples are not provided.

^a^Examples reflect the parent website of a search result. For example, the page of the University of Maryland Medical Center that appeared in the page was about acupuncture.

^b^CIH: complementary and integrative health.

**Table 6:** Frequencies of website types by domain (N=165).

Website type	Domain				
	Acupuncture (n=37), n (%)	Homeopathy (n=33), n (%)	Massage (n=31), n (%)	Reiki (n=37), n (%)	Yoga (n=27), n (%)
Web providers or portal sites	8 (21.6)	4 (12.1)	7 (22.6)	3 (8.1)	1 (3.7)
Support groups	0 (0)	0 (0)	0 (0)	0 (0)	0 (0)
Nonprofit organizations	1 (2.7)	4 (12.1)	1 (3.2)	1 (2.7)	1 (3.7)
Government websites	4 (10.8)	1 (3.0)	2 (6.5)	2 (5.4)	0 (0)
Sale of CIH^a^ services	10 (27.0)	4 (12.1)	10 (32.3)	15 (40.5)	11 (40.7)
Sale of CIH products	8 (21.6)	6 (18.2)	4 (12.9)	10 (27.0)	6 (22.2)
Personal sites^b^	6 (16.2)	6 (18.2)	5 (16.1)	10 (27.0)	5 (18.5)
Information services	16 (43.2)	7 (21.2)	7 (22.6)	5 (13.5)	0 (0)
Media sites	8 (21.6)	9 (27.3)	5 (16.1)	6 (16.2)	12 (44.4)
Clinician sites	10 (27.0)	5 (15.2)	6 (19.4)	14 (37.8)	7 (25.9)

^a^CIH: complementary and integrative health.

^b^Totals include the additional blogs that were added to each domain as specified in the “Methods” section.

#### Quality Assessment

**Figure 3:**
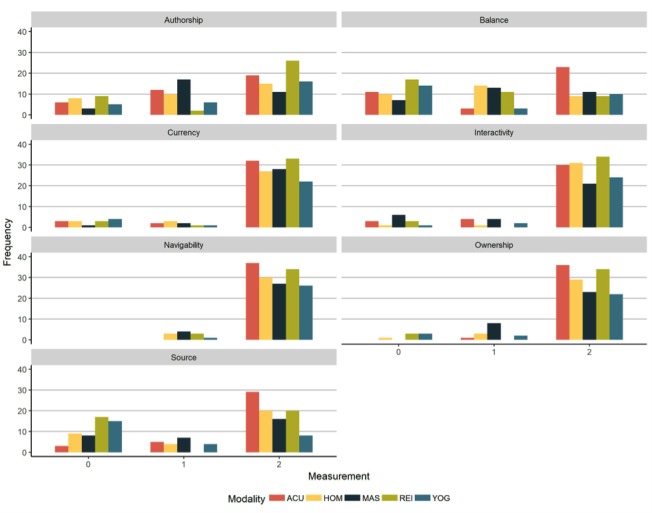
Sandvik quality indicators. ACU: acupuncture, HOM: homeopathy, MAS: massage, REI: reiki, YOG: yoga.

We first considered the websites based on the Sandvik scale. Across all domains, a high proportion of websites received a score of 2 on ownership, currency, interactivity, and navigability (Figure 3). Scores were more variable with respect to authorship, balanced presentation of information, and the use of sources of information. Acupuncture demonstrated a unique pattern in terms of balance of information, with a tendency for ratings to be at the two extremes: 0 (only promoting own products or services), and 2 (balanced information). There was also a greater tendency to include source attribution than would have been expected, had all domains been equal (*P*<.001; see Multimedia Appendix 2 for standardized Pearson residuals and Multimedia Appendix 3 for pairwise comparisons of source attribution).

### Aim 2: Message Content, Structural Features, and Presentation Style

**Figure 4:**
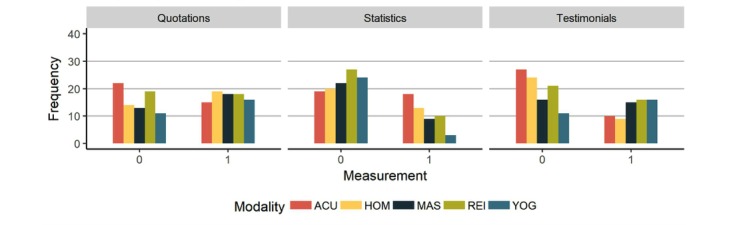
Message characteristics. ACU: acupuncture, HOM: homeopathy, MAS: massage, REI: reiki, YOG: yoga.

We also examined message content, structural features and presentation style of websites. With regard to message content, there was considerable consistency across domains. Websites were divided on the inclusion of statistics, testimonials and quotations (Figure 4). A notable exception was yoga websites’ paucity of statistics.

**Figure 5:**
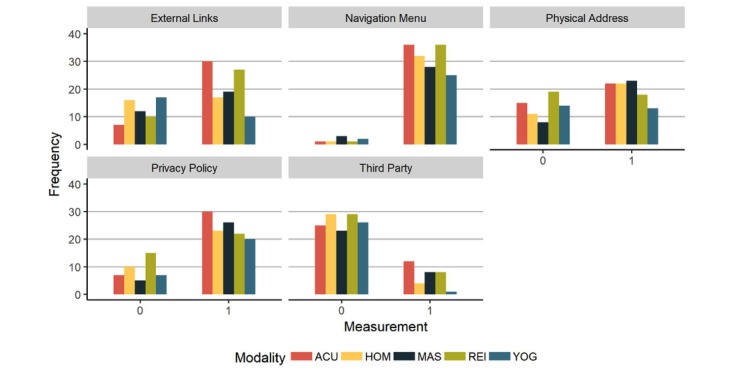
Structural characteristics. ACU: acupuncture, HOM: homeopathy, MAS: massage, REI: reiki, YOG: Yoga.

With regard to structural features, nearly all websites had navigation menus, and most had privacy policies (Figure 5). The presence of links to external websites differed across domains (N=165, Χ^2^_4_=16.4, *P*=.002) with acupuncture sites tending to include more external links, and yoga, fewer, based on standardized Pearson residuals greater than an absolute value of 2 (see Multimedia Appendix 2 for standardized Pearson residuals, and Multimedia Appendix 3 for pairwise chi-square tests of presences of external links). The presence of physical locations was more common than not among homeopathy and massage websites, and more evenly split in the case of the other domains. In general, websites did not have a third-party accreditation.

**Figure 6:**
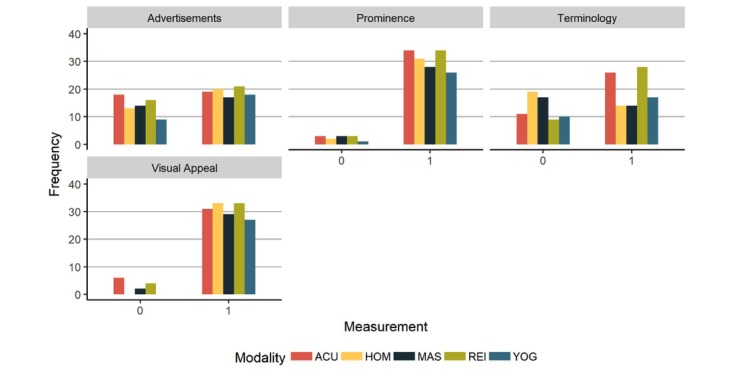
Presentation style. ACU: acupuncture, HOM: homeopathy, MAS: massage, REI: reiki, YOG: yoga.

We now consider presentation style of the websites. Almost all had CIH information prominently displayed and presented information in a visually appealing fashion (Figure 6). They were generally mixed in terms of whether they contained advertisements. There was variation across domains in the extent to which the websites contained domain-specific terminology (N=165, Χ^2^_4_=12.6, *P*=.01). Reiki was more likely to have been rated as having domain-specific terminology, homeopathy was less likely, based on standardized Pearson residuals of greater than an absolute value of 2 (see Multimedia Appendix 2 for standardized Pearson residuals, and Multimedia Appendix 3 for pairwise chi-square tests of presences of domain-specific terminologies).

### Aim 3: Harms, Benefits, and Website Purpose

We examined the extent to which websites reported harms and benefits (Table 7). The number of websites not providing information about harms was high (119/165, 72.1%), as was the number of websites stating that a given modality offered multiple benefits (90/165, 54.5%). Pain reduction and psychological benefits were also frequently mentioned, in 69 (41.8%) and 51 (30.9%) of websites, respectively. While almost all websites that were coded “no benefit” did not mention any benefits, there were a few cases, such as Wikipedia and Quackwatch, in which the websites stated that there were no benefits. The benefits have been described as categories, as opposed to specific harms, to be concise. Examples of other harms that were noted include pneumothorax, broken needle, organ puncture, pain, drowsiness, dizziness, contamination, soreness, flu-like symptoms, blood sugar irregularities, and muscle strain. Examples of other benefits included benefits for sleep, fatigue, energy, relaxation, stress, immune function, anti-aging, wellness, and more.

With regard to purposes, providing information (126/165, 76.4%) and sales (61/165, 37.0%) were the most common (Table 8). Sites that were identified as providing information to support the professional nature of the modality included (1) the British Acupuncture Council [57], (2) Acupuncture Board, an autonomous body under the umbrella of the Department of Consumer Affairs, which licenses and regulates acupuncturists in California [58], (3) the British Homeopathic Association [59], and (4) the International Association of Reiki Professionals [60].

**Table 7:** Prevalence of harms and benefits (N=165).

Category		n (%)
**Harms**		
	No harm	119 (72.1)
	Misinformation	12 (7.3)
	Improper	8 (4.8)
	Contamination	4 (2.4)
	Bleeding	3 (1.8)
	Bruising	3 (1.8)
	Syncope	1 (0.6)
	Other	26 (15.8)
**Benefits**		
	Multiple benefits	90 (54.5)
	Pain reduction	69 (41.8)
	Psychological benefit	51 (30.9)
	No benefit	48 (29.1)
	Digestive	29 (17.6)
	Neurologic	25 (15.2)
	Endocrine	20 (12.1)
	Gynecologic	12 (7.3)
	Cardiovascular	11 (6.7)
	Circulatory	10 (6.1)
	Posture	9 (5.5)
	Other	51 (30.9)

**Table 8:** Prevalence of website purposes (N=165).

Website purpose	n (%)
Providing information	126 (76.4)
Sales	61 (37.0)
Training	12 (7.3)
Professionalization	11 (6.7)
Quality	8 (4.8)
History	7 (4.2)
Promotion of research	6 (3.6)
Present learned consensus	1 (0.6)
Other	18 (10.9)

Many of these sites were also noted as providing information about the history of the modality and promoting research on the modality. Though it was observed earlier that there were few government websites, sites that provide information about the profession may serve as regulatory bodies and provide varying degrees of quality control; thus, these sites may complement the role served by government websites such as that of the NCCIH.

## Discussion

### Principal Findings

We employed a systematic search strategy to identify top search results across 5 CIH domains and then performed quantitative content analysis to characterize the nature, quality, and assertions made by these websites. The most common types of websites were those selling services, and clinician, media, information services, and product websites. Overall, the diverse distribution of website types suggests that, regardless of CIH domain, the public encounters information through many different types of media, and it is important to consider how the presentation of this content may differ depending on the medium.

Regarding the overall quality, we observed similar patterns across domains on specific dimensions but not others. Websites received high scores on ownership, currency, interactivity, and navigability, but were inconsistent with respect to authorship, balanced presentation of information, and source attribution. These results suggest that guidelines and other efforts to improve information quality relating to CIH should place greater emphasis on recommendations for these dimensions. Concerning message content, we again observed similar patterns across domains in that websites were split regarding their inclusion of statistics, testimonials, and quotations.

The findings for structural features and presentation style appeared to differ across domains. Websites generally had a navigation menu and privacy policy. They also included information on CIH topics in a prominent way and were visually appealing. The listing of a physical address was more common among homeopathy and massage websites. Acupuncture and reiki sites tended to include more external links, and yoga, fewer. Also, there was variation across domains in the extent to which the websites contained domain-specific terminology. A substantive portion of websites reported either multiple benefits or no benefits, and reporting of harms was scarce. Though information provision was an important purpose for most of the websites that we reviewed (126/165, 76.4%), sales were also an important function (61/165, 37.0%).

The characteristics for which variability exist are areas that warrant greater attention from researchers, policy makers, clinicians, and patients. On their own, these characteristics may not necessarily be positive or negative. For example, testimonials and quotations could potentially provide misleading information, but they could also help readers to better understand the nature of the services being offered and particularly for those with more experience, to distinguish between different entities offering the same service. Similarly, references to external material and domain-specific terminology can provide helpful information for individuals that are new to a modality, but they can also confuse readers if the explanatory text is insufficient. Thus, this review on its own is not intended to provide a clear set of guidelines to be followed, but rather, identify dimensions for which there is a need to develop guidelines and patient education materials, and to assist individuals in the consumption and evaluation of CIH content that is publicly available on the internet.

### Comparison With Prior Work

This study adds to the extant literature concerning CIH in multiple ways. First, the study provides a characterization of the types, quality, and other characteristics of websites providing CIH-related information that may influence how health consumers interact with these websites. Structural features and presentation style can be associated with perceptions of website credibility [43,61], and design can play an important role in credibility. Thus, the study provides a basic understanding of these websites and differences across CIH domains.

However, the study does not answer the question of how people might react to online CIH information. In this study, we found that websites providing CIH-related information often did not report harms and reported multiple benefits. Additionally, the Sandvik scores showed that the degree to which the information was balanced varied in the domains reviewed. However, individuals might be influenced regardless of the degree to which websites present balanced information, and even when presented with the same information, people may make different decisions.

There is a need to understand the variability in individuals’ decision-making processes concerning CIH, especially differences in how people may respond to online information about CIH. Previous research has reported that beliefs and attitudes are associated with CAM use [12,14], and some theoretical models have been used to conceptualize the process of choosing to utilize CAM [62]. However, none of these models have emphasized the role or presentation of information. Thus, more research is needed to understand how people interact with CIH-related websites and information, and how this influences their decisions to use CIH and their selection of modalities.

We might also consider the potential implications of the Elaboration Likelihood Model of Persuasion, which argues that individuals respond to source and message characteristics in different ways depending on the extent to which they focus on topics [63]. Prior research has also reported that individuals’ evaluation of, and interactions with, information can change over the course of a chronic condition [64,65]. Given that individuals might respond differently based on a combination of personal, situational and source factors, there is a need for additional research to understand how individuals may interact with the CIH information that they encounter online, and in turn, assist them to make informed decisions. This may be particularly important given that extant literature has reported that patients often do not report CIH use to their health care providers [17,66,67].

It may also be of interest to compare the results of this study to prior research on the quality of websites relating to particular conditions. Two conditions for which CIH use is common are chronic pain and inflammatory bowel disease [7,68]. Websites providing health information for these conditions have been shown to be variable in quality and possess shortcomings in terms of source attribution, links to additional information, and balanced reporting [69,70]. Thus, these issues are not necessarily unique to CIH. However, in a subject matter area where much remains to be understood, continual work to improve the quality of online information and efforts to educate patients to make informed decisions is vital.

Lastly, it is interesting to consider the full range of benefits that were mentioned in the online CIH-related content included in this review. Aside from pain reduction and psychological benefits, a wide variety of benefits including sleep, fatigue, energy, stress, immune function, and wellness were also mentioned. These findings are consistent with previous review literature. Extant studies have reported significant associations between CAM use and health factors including arthritis, anxiety or depression, cancer, diabetes, chronic conditions, psychological health, as well as self-rated general health [71]. Secondary analysis of National Health Interview Survey data has also illustrated the importance of wellness and of CAM as part of a self-management style, to CAM users [15].

### Limitations

This study had several limitations. First, our study was limited in the number of websites that was reviewed. Our search strategy and website inclusion criteria were similar to those that have been used in previous research on online health information quality; however, when an individual is searching for CIH information, it is likely that they will perform multiple searches, and thus be exposed to websites that were not included in our review. Second, the rankings provided by search engines are generated by “commercial” algorithms and are not necessarily consistent over time or place [72]. Third, in this study, we employed checklists for which there was a limited set of choices (0, 1, 2 or 0, 1). It is possible that if we had employed a more granular set of scales, then we would have seen more variability among websites.

Last, in this study, we selected 5 types of CIH to provide a richer and more comprehensive assessment of the diversity of online health information quality in CIH. Though we endeavored to select a diverse set of modalities, there is considerable diversity both within and across CIH domains, and thus our conclusions are limited insofar as the domains may be representative. CIH modalities vary in terms of many characteristics, including their degree of acceptance by the public, the ways in which modalities are utilized (eg, through the services of a provider, class or product), the degree to which provider associations exist and provide support to practitioners, and the extent to which evidence-based information is available. Considering the quality of online health information in the context of these characteristics could potentially be of considerable interest, but was beyond the scope of this study.

### Conclusion

In this study, we selected multiple domains of CIH and characterized them through multiple dimensions (1) type and quality, (2) message, structural, and presentation characteristics; and (3) harms, benefits and website purposes. To our knowledge, this is the first study to perform a multidimensional assessment of websites in multiple CIH domains. In considering CIH, it is important to realize that the term does not represent a single tradition. There are differences in modalities resulting from a myriad of factors including the history, conceptual foundations, level of recognition, service delivery models, and perceptions in the media. This review showed that while there are similarities among websites of different CIH domains, there are also differences. Professional associations and regulatory bodies for these different domains might use this study to develop guidelines that they could provide to practitioners. There are also characteristics on which websites tended to be split in terms of presence and prevalence, such as the provision of quotations, testimonials, external links, and terminology. While the presence or absence of such characteristics may not necessarily be a positive or a negative characteristic, the findings of this study could be helpful for practitioners to consider how to present information to their clients and patients, as well as to consumers, to assist them in evaluation of the content that they encounter. As such, this article might be used by a diverse audience for a variety of purposes (eg, by professional associations interested in developing guidelines for website development), CIH practitioners who are interested to learn about others in similar and different clinical care modalities, practitioners of allopathic medicine, and patients. A general understanding of some of the variability of website characteristics could help both health care providers and patients consider the websites that they encounter with greater discernment. Lastly, this article can assist researchers who are interested in understanding the content and manner of presentation of information that lay health consumers are exposed to about CIH, as well as to identify what we do not yet understand about the presentation of this information.

## Appendix

Multimedia Appendix 1 Interrater reliability.

Multimedia Appendix 2 Chi-square tests of independence for website characteristics.

Multimedia Appendix 3 Pairwise comparisons of quality assessments.

## Abbreviations

CAM: complementary and alternative medicine
CIH: complementary and integrative health
NCCIH: National Center for Complementary and Integrative Health
SERP: second search engine results page
