# Supplementary material for: Characterizing Websites That Provide Information About Complementary and Integrative Health: Systematic Search and Evaluation of Five Domains
Source: Interact J Med Res. 2018 Oct 10;7(2):e14. doi: 10.2196/ijmr.9803 (PMC6231734; doi:10.2196/ijmr.9803)
Supplement: Multimedia Appendix 1 [file ijmr_v7i2e14_app1.pdf]

Multimedia Appendix 1. Interrater reliability.

Domain	Kappa
Acupuncture	.87
Homeopathy	.76
Massage Therapy	.96
Reiki	.80
Yoga	.72
Across all domains	.82
