# Supplementary material for: Characterizing Websites That Provide Information About Complementary and Integrative Health: Systematic Search and Evaluation of Five Domains
Source: Interact J Med Res. 2018 Oct 10;7(2):e14. doi: 10.2196/ijmr.9803 (PMC6231734; doi:10.2196/ijmr.9803)
Supplement: Multimedia Appendix 2 [file ijmr_v7i2e14_app2.pdf]

Multimedia Appendix 2. Chi-square tests of independence for website characteristics.

A standardized Pearson residual that exceeds 2 in absolute value indicates a lack of fit of the model in a given cell [53,54].

Table 1. Domain versus source attribution.

Source attribution differed across domains, X^2(8**, *N***=165) = 28.71, *p* = 0.0004. Because there were expected frequencies less than 5, we also employed Fisher's Exact test (*p* = 5.73e-05).

Modality/Source Attribution	0			1			2		
	O	E	R	O	E	R	O	E	R
Acupuncture	3	11.66	-3.48	5	4.48	0.29	29	20.85	3.07
Homeopathy	9	10.4	-0.59	4	4.0	0	20	18.60	0.55
Massage Therapy	8	9.77	-0.76	7	3.76	1.98	16	17.47	-0.59
Reiki	17	11.66	2.15	0	4.48	-2.56	20	20.85	-0.32
Yoga	15	8.51	2.94	4	3.27	0.47	8	15.22	-3.06

Key: O = Observed, E = Expected, R = Residual.
2 = References are given to scientific literature.
1 = References are given to non-scientific sources.
0 = There is no indication of sources of information used to compose the content of the website.

Table 2. Domain versus presence of external links.

The presence of links to external websites differed across domains, X^2(4, *N*=165)=16.36, *p*=.003.

Modality/Source Attribution	0			1		
	Observed	Expected	Residual	Observed	Expected	Residual
Acupuncture	7	13.9	-2.66	30	23.1	2.66
Homeopathy	16	12.4	1.45	17	20.6	-1.45
Massage Therapy	12	11.65	0.14	19	19.35	-0.14
Reiki	10	13.9	-1.5	27	23.1	1.5
Yoga	17	10.15	2.98	10	16.85	-2.98

Key: 1= The website includes links to external websites
0 = The website does not include links to external websites

Table 3. Domain versus presence of domain-specific terminology.

There was variation across domains in the extent to which the websites contained domain-specific terminology, $X^2(4, N=165)=12.61$, $p=.01$.

Modality/Source Attribution	0			1		
	Observed	Expected	Residual	Observed	Expected	Residual
Acupuncture	11	14.8	-1.45	26	22.2	1.45
Homeopathy	19	13.2	2.3	14	19.8	-2.3
Massage Therapy	17	12.4	1.87	14	18.6	-1.87
Reiki	9	14.8	-2.21	28	22.2	2.21
Yoga	10	10.8	-0.34	17	16.2	0.34

Description: Use of CIH language and terms specific to that domain (e.g., “chi” or “Qi” - acupuncture, "like cures like" - homeopathy, "friction" - massage therapy, "Ki" - Reiki, "prana" - yoga)
Key: 1 = The website contains domain-specific terminology
0 = The website does not contain domain-specific terminology
