# Supplementary material for: Characterizing Websites That Provide Information About Complementary and Integrative Health: Systematic Search and Evaluation of Five Domains
Source: Interact J Med Res. 2018 Oct 10;7(2):e14. doi: 10.2196/ijmr.9803 (PMC6231734; doi:10.2196/ijmr.9803)
Supplement: Multimedia Appendix 3 [file ijmr_v7i2e14_app3.pdf]

Multimedia Appendix 3. Pairwise comparisons of quality assessments.

Table 1. Pairwise chi-square tests of source attribution.

Domain#1	Domain#2	*p*-value	Adj. *p*-value (Hommel)
Acupuncture	Homeopathy	0.11	0.12
	Massage	0.04	0.07
	Reiki	0.0001	0.001
	Yoga	4.01e-05	0.0004
Homeopathy	Massage	0.58	0.58
	Reiki	0.04	0.07
	Yoga	0.05	0.07
Massage	Reiki	0.005	0.02
	Yoga	0.07	0.09
Reiki	Yoga	0.02	0.05

Table 2. Pairwise chi-square tests of presences of external links.

Domain#1	Domain#2	***p*-value**	**Adj. *p*-value (Hommel)**
Acupuncture	Homeopathy	0.01	0.04
	Massage	0.10	0.19
	Reiki	0.58	0.58
	Yoga	0.0006	0.01
Homeopathy	Massage	0.46	0.51
	Reiki	0.08	0.19
	Yoga	0.30	0.44
Massage	Reiki	0.44	0.51
	Yoga	0.11	0.19
Reiki	Yoga	0.01	0.03

Table 3. Pairwise chi-square tests of presences of domain-specific terminologies.

Domain#1	Domain#2	***p*-value**	**Adjusted *p*-value (Hommel)**
Acupuncture	Homeopathy	0.03	0.10
	Massage	0.05	0.122
	Reiki	0.79	0.88
	Yoga	0.60	0.75
Homeopathy	Massage	1.00	1.00
	Reiki	0.01	0.06

	Yoga	0.13	0.26
Massage	Reiki	0.01	0.06
	Yoga	0.20	0.33
Reiki	Yoga	0.29	0.41
